# Machiavellianism and learning-related subjective well-being among Chinese senior high school students: A moderated mediation model

**DOI:** 10.3389/fpsyg.2022.915235

**Published:** 2022-09-29

**Authors:** Minqi Yang, Chunyu Qu, Hanxiao Guo, Xicheng Guo, Kexin Tian, Guofang Wang

**Affiliations:** ^1^School of Education, Zhengzhou University, Zhengzhou, China; ^2^School of Sociology, China University of Political Science and Law, Beijing, China

**Keywords:** Machiavellianism, learning subjective well-being, gratitude, subjective family economic level, senior high school students

## Abstract

Based on the life history theory and broadening construction theory, the study aimed to investigate the influence of Machiavellianism on the learning-related subjective well-being and the underlying mechanism, 582 Chinese senior high school students (16.8 ± 0.9 years old) including 289 girls (48.3%) and 310 boys were recruited to participate in this study, and they anonymously filled out questionnaires regarding Machiavellianism, learning-related subjective well-being, gratitude, and subjective family economic level. The results showed that: (1) a higher level of Machiavellianism was associated with a lower level of learning-related subjective well-being; (2) gratitude partially mediated the relationship between Machiavellianism and learning-related subjective well-being; (3) subjective family economic level moderated the links between Machiavellianism and learning-related subjective well-being, and between gratitude and learning-related subjective well-being. This study explained how and when Machiavellianism affected Chinese senior high school students’ learning-related subjective well-being and provided a deeper understanding of the relationship between Machiavellianism and learning-related subjective well-being.

## Introduction

Subjective well-being (SWB), including happiness, life satisfaction, and positive affect (Diener, 2009), is defined that people making an evaluation of the general quality of life-based on their own standards (Suh et al., 1996), reflecting the individual adaptive status and cognitive evaluation to the social environment (Andrews and McKennell, 1980). Learning-related subjective well-being is the embodiment of subjective well-being in the learning process. Specifically, it refers to a sense of satisfaction, achievement, and pleasure that students make comprehensive evaluations of learning based on personal standards in the three evaluation stages of pre-learning, during learning, and after learning (Gan, 2010). In China, students, especially senior high school students, are experiencing huge pressure to get higher education. The heavy academic burden and high expectations from the parents make students experience anxiety, loneliness, and depression (Liao, 2013; Shi et al., 2013; Li and Zhang, 2014; Peng et al., 2021), which leads to serious consequences such as suicide (Li, 2020; Shen et al., 2021; Zhang et al., 2021). Therefore, the learning-related subjective well-being of senior high school students should be taken seriously. Additionally, in July 2021, the Chinese Ministry of Education released the “Double Reduction Policy,” which is committed to reducing students’ burden, relieving students’ pressure, and promoting the healthy growth of students. This also makes the research on the learning-related subjective well-being of high school students urgent.

Machiavellianism is often used to describe individuals who manipulate and exploit others for their purposes (Geng et al., 2015). It is characterized by a cynical view of human nature, a belief in the effectiveness of manipulative tactics in social interaction, and a moral outlook that puts self-interest above principle (Christie and Geis, 1970; Rauthmann, 2011; Cohen, 2016). The relationship between the Dark Triad Traits (i.e., Machiavellianism, psychopathy, and narcissism) and well-being has been well-documented (Aghababaei and Błachnio, 2015; Ma et al., 2021; Van Groningen et al., 2021). For example, Joshanloo (2021) and Rehman et al. (2018) found that Machiavellianism was negatively associated with well-being. And Machiavellianism is not only positively correlated with depression and perfectionism (Sherry et al., 2006; Bianchi and Mirkovic, 2020), but also negatively affects individuals’ life satisfaction, subjective well-being (Sherry et al., 2006), and motivation for happiness (Cai et al., 2017). There is also an empirical study indicating that among all five empirically derived Dark Triad profiles, students with the low Machiavellianism profile exhibited the highest probability of “flourishing” (Hu and Lan, 2022). Furthermore, according to life history theory (LHT; Figueredo et al., 2006), which is a mid-level theory from evolutionary biology, individuals must make a balance between survival efforts and reproduction efforts to adapt to the environment. Specifically, if energy and resources are allocated to survival, individuals will pay attention to maintaining their bodies, developing knowledge and skills, raising offspring, and having long-term plans. On the contrary, if the energy and resources are allocated to reproduction, the individual tends to show precocious puberty, have more children and less investment in raising their generations, and have a preference for immediate satisfaction and short-term benefits (McDonald et al., 2012; Yang et al., 2022). The former can be called “slow life history strategy,” while the latter can be called “fast life history strategy” (Jonason et al., 2015). These two strategies constitute the two poles of the life history strategy continuum. The individuals’ balancing results determine their position in the continuum and lead to the formation of corresponding personality traits. As an executive agency, the personality traits coordinate behavior and environment to work together to complete adaptation tasks (Geng et al., 2014; Birkás et al., 2020). Both the fast life history strategy and the slow life history strategy have adaptive significance, but individuals holding the fast life history strategy solve their adaptation problems at the cost of physical and mental health and quality of life. Studies showed that Machiavellianism is one of the indicators of the fast life history strategy (Crysel et al., 2013; Jonason et al., 2015). Thus we hypothesized that Machiavellianism would be negatively correlated with learning-related subjective well-being (H1).

Gratitude is one of the most important virtues developed by human beings in the process of evolution and is defined as the emotional experience or state of mind that an individual produces when he receives a certain kind of favor (Zhang and Hou, 2010). According to the broadening construction theory of gratitude (Fredrickson and Joiner, 2002), gratitude has the function of inducing positive emotion. Previous studies have shown that gratitude is a valid predictor of happiness, stress perception, and depression (Xie et al., 2013; He et al., 2015). According to the positive emotion expansion construction theory proposed by Fredrickson, gratitude could help individuals to expand cognitive schemas, enhance thinking flexibility, organize social capital, and eliminate the negative effects caused by negative emotions (Fredrickson and Joiner, 2002). And the achievement motivation theory (Anderman, 2020) points out that gratitude can enhance the motivation for goal pursuit, promote teenagers’ academic participation, and then improve their happiness. Researchers have found that individuals with a higher level of gratitude have a higher quality of mental health (Deng et al., 2016; Ding and Zhao, 2018; Li and Zhou, 2018). Many empirical studies have demonstrated that trait gratitude is positively related to SWB (Froh et al., 2008; Wood et al., 2010; Alkozei et al., 2018; Chopik et al., 2019). For instance, a longitudinal study found that trait gratitude could predict the positive aspects of SWB (i.e., life satisfaction and positive affect) in Chinese adolescents (Yang et al., 2021). Some studies have also found that the higher the level of individual gratitude, the easier it is to obtain social support in the face of difficulties, which can improve their subjective well-being (Luo and Zhou, 2015). Given that, we speculated that the level of gratitude of students could be positively correlated with learning-related subjective well-being (H2).

According to the implicit gratitude theory, gratitude is an unconscious or automatic emotional response, which attempts to return kindness to others based on acknowledging the kindness or help from the benefactors (He et al., 2013). Whereas Machiavellianism can hinder individuals from experiencing the feeling of gratitude and further trigger their exploitative behavior (Arathy et al., 2021). In addition, in terms of moral and psychological judgment, Machiavellians do not feel favored by others, so it is difficult to translate into gratitude practice (Qin, 2013). Given the findings outlined above, we predicted that gratitude could mediate the relationship between Machiavellianism and learning-related subjective well-being (H3).

Subjective well-being is not an ungrounded subjective feeling, it is affected by economic conditions (Hou et al., 2009; Yao et al., 2015). Researchers have found that family economic level and income status could directly affect the subjective well-being of individuals (Yang et al., 2015). In addition, researchers also demonstrated that economic status moderated the effects of the predictive factors on subjective well-being. For example, income plays a moderating role in the relationship between job satisfaction and subjective well-being in urban China (Nielsen et al., 2011) and between social relationships and subjective well-being (Mullen et al., 1989). A meta-analysis has indicated that subjective well-being has a stronger link to socioeconomic status measured subjectively as relative rank than measured objectively as income or educational attainment (Tan et al., 2020). Studies have revealed that family subjective socioeconomic status is a strong and positive factor that contributes to well-being, demonstrating that individuals with higher family subjective socioeconomic status exhibited greater well-being (Navarro-Carrillo et al., 2020; Yan et al., 2022). Thus, we predicted that subjective family economic level could moderate the links between learning-related subjective well-being and Machiavellianism and gratitude (H4).

Taken together, the current study aimed to explore the relationship between Machiavellianism and learning-related subjective well-being among Chinese senior high school students and to investigate the mediating effect of gratitude and the moderating effect of subjective family economic level as well.

## Materials and methods

### Participants

The final data was collected from 582 (the sample size would provide sufficient power according to justifications for the sample size in a study (Daniël, 2022) and the priori sample size (*n* = 105) calculation conducted *via* Gpower [effect size was set at 0.2 (medium), *α* = 0.5, *β* = 0.95)] senior high school students in Henan, China in September 2021. The participants, with a mean age of 16.8 (SD = 0.9) years, included 289 girls (48.3%) and 310 boys (51.7%). Among these participants, 347 were from Grade 12, 176 from Grade 11, and 72 from Grade 10. The study was approved by the Ethics Committee of the institution and carried out following the approved guidelines and regulations. The written informed assent was obtained from the participants and the written informed consent was obtained from the student’s guardians. And all participants were paid for their participation.

### Measures

*Machiavellianism* was measured with the self-report and validated Chinese version of Kiddie Machiavellian Scale revised by Geng et al. (2011). The scale is composed of 16 items that assess distrust (five items; e.g., “*Sometimes you have to hurt others in order to get what you want*”), distrust of humanity (seven items; e.g., “*Do not tell anyone the real reason for doing something unless you have a special purpose*”), and dishonest (four items; e.g., “*The best way to interact with someone is to say what they want to know*.”). Participants answered on a 4-point Likert scale, ranging from 0 (total agreement) to 3 (total disagreement). The higher scores indicated lower levels of Machiavellianism. In this study, Cronbach’s α coefficient is 0.71.

*The learning-related subjective well-being* was evaluated with the Learning-related Subjective Well-being Questionnaire developed by Ma and Liu (2005). The questionnaire is consisted of 18 items, including learning achievement (six items; e.g., “*I can study with high energy*”), positive learning experience (four items; e.g., “*I can often achieve my goals*”), learning values (four items; e.g., “*I am so bad at learning that I want to give up*”), and negative experiences of learning (four items; e.g., “*I feel empty lately and do not know what to do*”). Each item was rated on a 5-point Likert scale (1 = “*not at all*,” 5 = “*completely*”). The score for each factor is generated by adding the scores of items within that factor. The Cronbach’s alpha coefficient for the total scale was 0.850, and the Cronbach’s alpha coefficients were 0.807 for academic achievement, 0.830 for the positive learning experience, 0.720 for learning value, and 0.722 for a negative learning experience.

*Gratitude* was measured with the Chinese version of the Gratitude Questionnaire (GQ-6; Wei et al., 2011), which contains six items, such as “*There are so many things in life for which I feel grateful.*” Each item was rated on a 7-point Likert scale (1 = “*strongly disagree*,” 5 = “*strongly agree*”). The total score is generated by adding the scores of all the items, with higher scores indicating higher levels of gratitude. In this study, Cronbach’s alpha coefficient for this questionnaire was 0.854.

*The subjective family economic level* was evaluated with the question “*How do you think about the economic level of your family?*,” which was adapted from the item “Family Economic Status” in the Postgraduate Stress Questionnaire (Yu and Zheng, 2005). Answers were recorded on a 5-point scale (1 = “*poor*,” 5 = “*rich*”) with higher scores indicating a higher subjective family economic level. When an item clears the conceptual ambiguity and reflects the common of one concept to participants’ daily experiences, single-item measures can be appropriated (Wanous and Reichers, 1996; Wanous et al., 1997; Cureu et al., 2021). And we had explained the meaning of economy in the item description, so we hope we could obtain an accurate indicator of this variable.

### Data analysis

Pearson correlation analysis was used to study the correlation of the variables (Machiavellianism, gratitude, household economic level). Mediated and chained mediator models were analyzed using the PROCESS macro in SPSS, with 95% confidence intervals that do not contain zero indicating a significant mediating effect. To determine whether the indirect or total effects were statistically significant, we used maximum likelihood estimates and bias-corrected bootstrap 95% confidence intervals based on 5,000 bootstraps and employed Model 15 to investigate the moderated mediation effects.

## Results

### Common method bias test

The Harman one-way test was used to test for common method bias (Podsakoff et al., 2003). The results showed that a total of 12 factors had characteristic roots greater than one and the variance explained by the first factor was 20.62%, which was less than 40%, indicating that there was no severe common method bias in this study.

### Preliminary analyses

Means, standard deviations, and correlations for all variables were presented in Table 1. Pearson’s correlation analysis results showed that learning-related subjective well-being was significantly and positively correlated with levels of gratitude, but negatively correlated with the level of Machiavellianism which was negatively correlated with gratitude, but positively associated with subjective family economic level.

**Table 1:** Descriptive statistics and correlations of the study variables.

	M ± SD	1	2	3	4	5	6	7	8	9	10	11
1 Learning-related subjective well-being	58.4 ± 12.5	1										
2 Academic achievement	15.4 ± 5.3	0.79^**^	1									
3 Positive experience of learning	12.9 ± 4.0	0.86^**^	0.53^**^	1								
4 A sense of the value of learning	16.4 ± 3.4	0.71^**^	0.32^**^	0.59^**^	1							
5 Negative experience of learning	13.8 ± 3.4	0.73^**^	0.41^**^	0.59^**^	0.37^**^	1						
6 Machiavellianism	52.3 ± 6.7	0.33^**^	0.21^**^	0.28^**^	0.28^**^	0.30^**^	1					
7 Distrust	13.3 ± 3.8	0.19^**^	0.08^*^	0.18^**^	0.21^**^	0.19^**^	0.75^**^	1				
8 Lack of faith in humanity	26.2 ± 3.5	0.34^**^	0.25^**^	0.28^**^	0.24^**^	0.28^**^	0.75^**^	0.22^**^	1			
9 Dishonesty	13.0 ± 2.0	0.14^**^	0.11^**^	0.10^**^	0.12^**^	0.13^**^	0.59^**^	0.19^**^	0.34^**^	1		
10 Gratitude	34.0 ± 7.1	0.39^**^	0.23^**^	0.32^**^	0.41^**^	0.27^**^	0.34^**^	0.23^**^	0.38^**^	0.05	1	
11 Subjective family economic level	3.2 ± 1.7	−0.05	−0.05	−0.03	−0.06	−0.03	−0.10^*^	−0.07	−0.08^*^	−0.06	−0.07	1

* *p* < 0.05. and

** *p* < 0.01.

### Mediating role of gratitude

First, this study used Model 4 in the PROCESS program developed by Hayes (2017) to test the mediating effect of gratitude in the association between Machiavellianism and learning-related subjective well-being. A mediation model with learning-related subjective well-being as the dependent variable, Machiavellianism as the independent variable, and gratitude as the mediating variable was conducted using Bootstrap’s method. The results showed the indirect effect was 0.10 with a 95% confidence interval of [0.065, 0.154], which does not contain 0, indicating a significant indirect effect of gratitude, which demonstrates the mediating role of gratitude. Besides, the direct effect of Machiavellianism on learning-related subjective well-being was 0.21 with a 95% confidence interval of [0.135, 0.288], not containing 0, which indicated that the direct effect of Machiavellianism was also significant, gratitude played a partial mediating role in the association between Machiavellianism and learning subjective well-being (Table 2).

**Table 2:** The direct effect and indirect effect in the mediating model.

	Effect	Boot SE	Boot CI lower 95%	Boot CI upper 95%	Relative effect values
Direct effect	0.21^**^	0.039	0.1348	0.2876	66.35%
Indirect effect	0.10^**^	0.022	0.0651	0.1539	33.65%
Overall effect	0.31^**^	0.0386	0.2424	0.3941	

** *p* < 0.01.

### Moderating role of subjective family economic level

**Figure 1:**
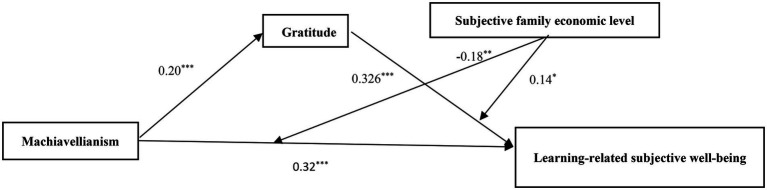
The mediating model for Machiavellianism and learning-related subjective well-being. ^*^p < 0.05, ^**^p < 0.01 and ^***^p < 0.001.

Using PROCESS 15, all variables were standardized and the equation model was shown in Table 3. The results showed the Machiavellianism was significantly predictive of subjective well-being of learning (*β* = 0.32, *p* < 0.001) and gratitude (*β* = 0.20, *p* < 0.001), gratitude was a positive predictor of learning-related subjective well-being (*β* = 0.32, *p* < 0.001), family economic level and Machiavellianism interaction term was a negative predictor of learning-related subjective well-being (*β* = −0.18, *p* < 0.01), and family economic level and gratitude interaction term positively predicted learning-related subjective well-being (*β* = 0.14, *p* < 0.05; Figure 1).

**Table 3:** Test the moderated mediation effect.

	Learning-related subjective well-being				Gratitude				Learning-related subjective well-being		
	*β*	*SE*	*t*		*β*	*SE*	*t*		*β*	*SE*	*t*
Machiavellianism	0.32	0.84	8.56^***^		0.21	0.04	5.43^***^		0.20	0.04	5.25^***^
Gratitude					0.33	0.04	8.07^***^		0.32	0.04	7.63^***^
Subjective family economic level									−0.17	0.07	−2.49^**^
Subjective family economic level*Machiavellianism									−0.18	0.06	−2.95^**^
Subjective family economic level* Gratitude									0.14	0.07	2.00^*^
*R^2^*		0.12			0.20					0.22	
*F*		72.51^***^			70.40^***^					30.68^***^	

* *p* < 0.05,

** *p* < 0.01

and ***p<0.001.

**Figure 2:**
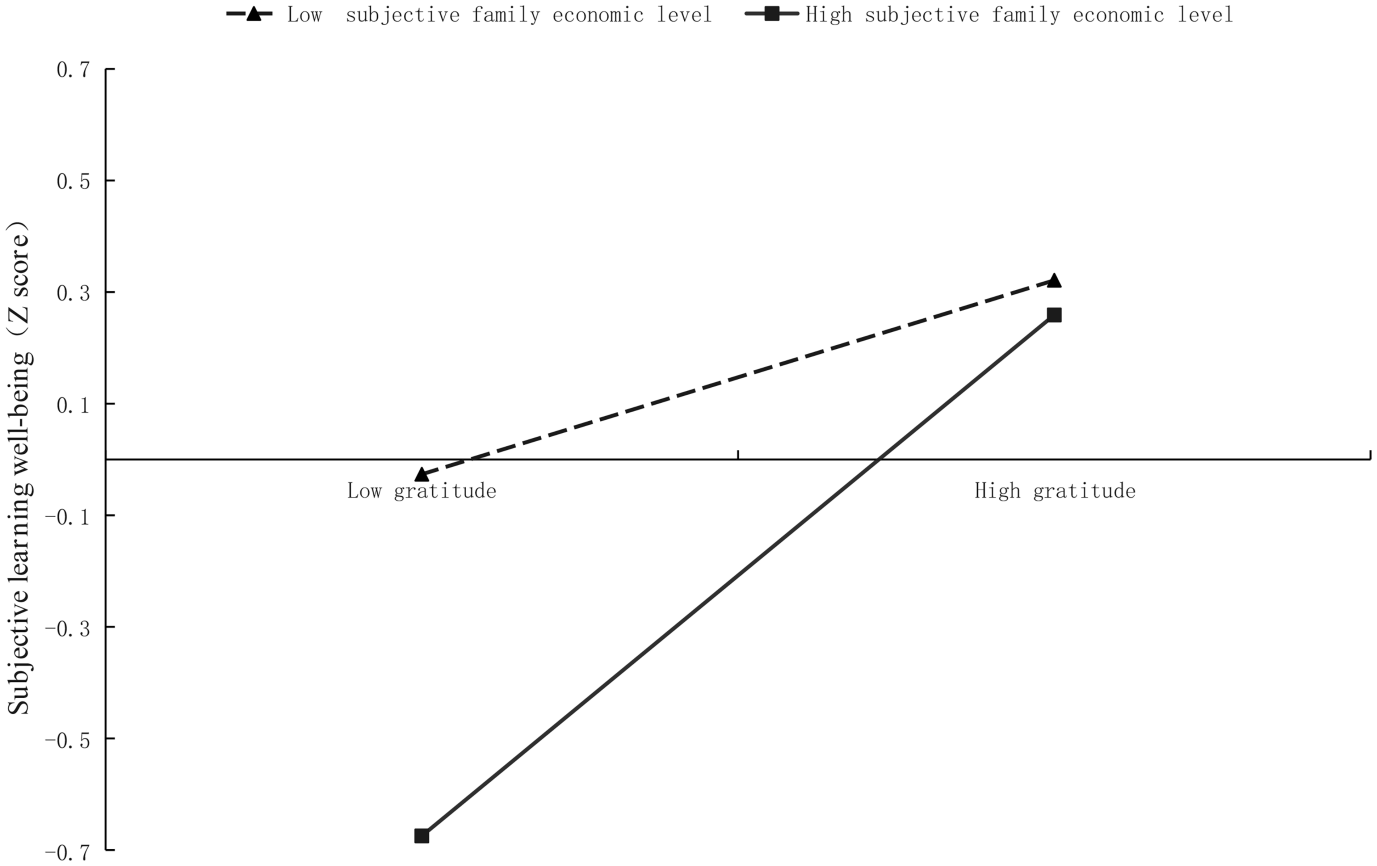
The interaction effect of subjective family economic level and gratitude on learning-related subjective well-being.

To further reveal how subjective family economic level moderated the effects of Machiavellianism and gratitude on the learning-related subjective well-being, the mean score of subjective family economic level plus or minus one standard deviation was divided into two groups of high and low subjective family economic level and the moderating effects were plotted. As shown in Figure 2, subjective family economic level positively moderated the relationship between gratitude and learning-related subjective well-being. Specifically, the predictive effect of gratitude on learning-related subjective well-being was stronger among students with high subjective family economic level [β_simple_ = 0.15, 95% CI (0.062, 0.240)] than among students with low subjective family economic level [β_simple_ = 0.058, 95% CI (0.008, 0.130)], which indicated higher subjective family economic level enhanced the positive effect of gratitude on learning-related subjective well-being.

**Figure 3:**
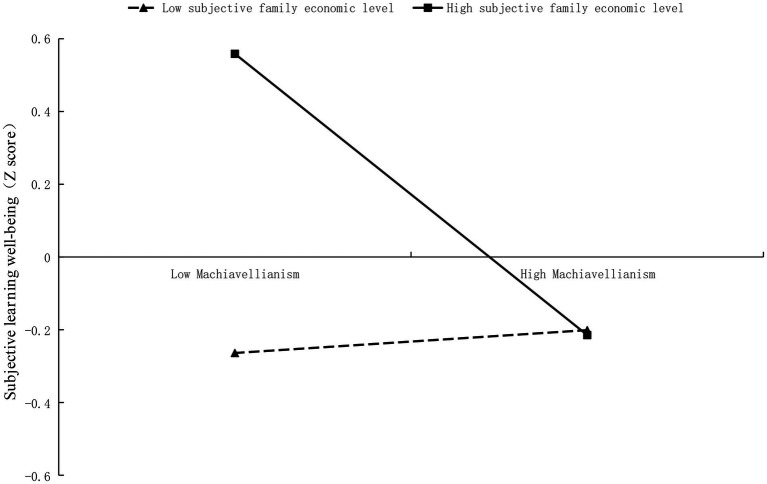
The interaction effect of subjective family economic level and Machiavellianism on learning-related subjective well-being.

As shown in Figure 3, subjective family economic level, however, negatively moderated the relationship between Machiavellianism and learning-related subjective well-being. Specifically, the negative predictive effect of Machiavellianism on learning-related subjective well-being was stronger among students with high subjective family economic level [*β*_simple_ = 0.40, 95% CI (0.247, 0.554)] than among the students with low subjective family economic level [*β*_simple_ = −0.01, 95% CI (−0.167, 0.154)], which indicated higher subjective family economic level buffered the negative effect of Machiavellianism on the learning-related subjective well-being.

## Discussion

The current study explored the relationship between Machiavellianism and learning-related subjective well-being of Chinese senior high school students, the mediating role of gratitude, and the moderating role of subjective family economic level, which was helpful to understand the psychological mechanism for the formulation of learning-related subjective well-being and had implications for enhancement of the learning-related subjective well-being of senior high school students. Consistent with the hypotheses, the results showed that the higher level of Machiavellianism was associated with the lower learning-related subjective well-being of senior high school students, gratitude played a partial mediating role between Machiavellianism and learning-related subjective well-being, and subjective family economic level moderated the effect of Machiavellianism and gratitude on learning-related subjective well-being.

The results of the current study showed that Machiavellianism was significantly negatively correlated with high school students’ learning-related subjective well-being, which was essentially consistent with previous studies. For example, Machiavellianism has a negative effect on teenagers’ motivation for happiness and well-being (Cai et al., 2017). Besides, the result also supported the life history theory (LHT), individuals holding the fast life history strategy with Machiavellianism being one of the indicators (Crysel et al., 2013; Jonason et al., 2015) tend to solve their adaptation problems at the cost of physical and mental health and quality of life. In the present study, to solve their adaptation problems, students high in Machiavellianism, who lack trust in others and have a higher level of interpersonal exclusion (He et al., 2018; Bloxsom et al., 2021), exploit others for short-term benefits and are difficult to obtain stable social support (Bloxsom et al., 2021). If things go on like this, although it helps to fulfill the short-term learning tasks, it will affect their learning motivation (Song et al., 2010), and reduce their positive learning experience (Kämmerle et al., 2014). Therefore, the higher the level of Machiavellianism of senior high school students, the lower their learning-related subjective well-being.

The current result also showed that gratitude had a positive impact on teenagers’ learning-related subjective well-being, which was consistent with a previous study showing that once gratitude is induced, individuals will have a sense of high learning efficacy and academic achievement (Hou, 2018). In addition, after experiencing negative events, individuals can maintain psychological balance through gratitude and eliminate the negative effects caused by negative emotions (Yang and Ye, 2014; Asici and Sari, 2021). The present study also showed that gratitude played a partial mediating role in the relationship between Machiavellianism and learning-related subjective well-being. Implicit gratitude theory goes that gratitude is an unconscious or automatic emotional response, which attempts to return kindness to others based on acknowledging the kindness or help from the benefactors (He et al., 2013). However, the personality traits of low empathy, such as Machiavellianism, are difficult to allow individuals to have positive feelings and empathy for the outside world (Aghababaei et al., 2018). Another study indicates that when encountering negative events in the learning process, individuals high in Machiavellianism could recognize less positive emotions and experience lower social support (Cheng and Li, 2010). Therefore, senior high school students with a high level of Machiavellianism have a lower level of gratitude, leading to lower learning-related subjective well-being.

The results of this study showed that subjective family economic level positively moderated the relationship between gratitude and learning-related subjective well-being, and negatively moderated the relationship between Machiavellianism and learning-related subjective well-being. Specifically, a higher subjective family economic level could enhance the positive effect of gratitude on the level of learning-related subjective well-being, and could also boaster the negative impact of Machiavellianism on the level of learning-related subjective well-being, but the lower subjective economic level weakened the associations between learning-related subjective well-being and Machiavellianism and gratitude. The results may be interpreted by LHT, that is, students from lower economic status are not so sensitive either to Machiavellianism or gratitude in shaping their well-being.

The result that the higher subjective economic level made learning-related subjective well-being more negatively associated with Machiavellianism, but more positively with gratitude, supported the resource amplification or “Matthew effect” hypothesis which holds that structural characteristics such as a higher socioeconomic status (SES, income is one important indicator of SES; Duncan et al., 2017) and individual characteristics coalesce in producing life outcomes (Walberg and Tsai, 1983) and were consistent with prior studies showing that the relationships between Conscientiousness and academic achievement were higher in higher- vs. lower-SES students (Brandt et al., 2020; Lechner et al., 2021).

Besides, individuals with high extraversion/positive emotionality/approach behavior which are closely associated with extraversion, are particularly sensitive to positive emotions and rewarding stimuli, whereas individuals with high neuroticism/negative emotionality/avoidance behavior which are closely associated with neuroticism, are particularly sensitive to negative emotions and stressful conditions (Watson and Clark, 1992; Watson et al., 1999; Canli et al., 2002; Canli, 2004). And Machiavellianism was positively correlated with neuroticism, and negatively with agreeableness, conscientiousness, and openness (Muris et al., 2013), which reflects negative emotionality and is sensitive to negative stimuli, whereas gratitude is positively associated with extraversion, and negatively correlated with neuroticism (McCullough et al., 2001), which reflects positive emotionality and is sensitive to positive stimuli. Thus learning-related subjective well-being could be negatively associated with Machiavellianism, but positively associated with gratitude even among the population from families with higher subjective economic levels.

What’s more, money has been called a “flexible and broadly useful coping resource” (McGonagle & Kessler, 1990, p. 697). Higher SES confers psychological advantages, such as higher levels of self-efficacy and coping abilities (Lorant et al., 2003). And a high SES is an important factor that could preserve reserve capacities and mitigate the progression of functional limitation (House et al., 2005; Singh-Manoux et al., 2005). According to the Family investment model (Conger and Donnellan, 2007), families with higher SES can provide a good educational and living environment and high-quality educational opportunities. Thus higher economic level could facilitate students with gratitude to benefit more regarding the learning-related subjective well-being.

Having higher economic level is not always a panacea, empirical studies showed that there was a negative relationship between the Dark Triad traits (Machiavellianism, psychopathy, and narcissism) and intellectual humility, and this relationship was stronger for privately schooled students, because high-SES individuals tend to be greedier, more unempathic and motivated by personal goals (Manstead, 2018), and are often overconfident and overestimate their ability more than others (Belmi et al., 2020). Besides, high subjective SES may also operate to exacerbate a sense of vulnerability (Wang and Liu, 2021). One study demonstrated that individuals with high subjective status showed significant cortisol increases when faced with social-evaluative threats (Gruenewald et al., 2006). According to the General Strain Theory (Agnew, 1992), individuals exposed to strains and stressors could develop negative emotions. Youth from higher SES backgrounds may experience additional stressors due to parental expectations and pressures to achieve as well as isolation or lack of parental involvement (Luthar and Latendresse, 2005). For example, individuals with high SES may worry that they fail to achieve goals valued by their family members (e.g., obtaining a university degree), and they may also be particularly likely to engage in conflicts with their family members (e.g., parents may have different values and feel disappointed), whereas, lower SES young people are more likely to have lower career self-efficacy and set lower educational and career aspirations (Thompson, 2013), and teachers have been shown to harbor lower expectations of less advantaged students (Tenenbaum and Ruck, 2007). What’s more, Machiavellians are well known to view others in a goal-oriented manner and tend to behave manipulatively to exploit others; that is, they see people ‘as a means to an end’ (Christie and Geis, 1970; Yang et al., 2019), Specifically, they give less importance to relationships, are ready to manipulate these for personal gains, have less trust on others, thus having more negative feelings from relationships (Gondal et al., 2020). That is to say, individuals higher in Machiavellianism may not only get pressure from their parents due to the higher economic level but also have negative emotions from the bad relationship among peers due to manipulative behaviors, thus the higher subjective economic level could exacerbate the negative association between Machiavellianism and learning-related subjective well-being.

There are some limitations of the current study that should be considered. Firstly, only self-report measures were used, and therefore the present study may be subject to mono-informant biases. Future research can combine multiple methods (such as simultaneously collecting data from the participant’s family members, teachers, and peers) and evidence from multiple indicators (such as behavioral studies, EEG, etc.) for further research. Secondly, the cross-sectional study design limits causal inferences, which is accompanied by a conceptual issue with the mediation model. That is, whether Machiavellianism or learning-related subjective well-being is the dependent variable is an arbitrary choice since Machiavellianism could also be the consequence of diminished learning-related subjective well-being based on the life history theory. Thus a longitudinal research design to investigate the causal relationship between the two variables is needed. Finally, this study only sampled high school students, which limits the generalizability of the results to the population. Future research should consider a more diverse population, such as primary school students and junior high school students.

## Conclusion

The results of this study revealed the effects of Machiavellianism on the Chinese senior high school students’ learning-related subjective well-being and the underlying mechanism: (1) The higher the level of Machiavellianism, the lower the learning-related subjective well-being of Chinese senior high school students; (2) Machiavellianism is not only directly associated with learning-related subjective well-being, but also indirectly associated with learning-related subjective well-being *via* the level of gratitude; (3) A higher subjective family economic level can enhance the positive effect of gratitude on the level of learning-related subjective well-being, but also could exacerbate the negative impact of Machiavellianism on the level of learning-related subjective well-being.

The current findings have important implications for improving senior high school students’ learning-related subjective well-being. First of all, Machiavellianism could have a direct and indirect effect *via* gratitude on learning-related subjective well-being, suggesting that the preventions and interventions for lowering the level of Machiavellianism and enhancing gratitude could both help to improve high school students’ learning-related subjective well-being. Secondly, the present findings suggest that a higher subjective family economic level could enhance the positive effects of gratitude, but boast the negative effects of Machiavellianism, on the learning-related subjective well-being, suggesting that a higher subjective family economic level could represent a “double-edged” sword, interventions for shaping the concept of money should take the gratitude and Machiavellianism into considerations. Finally, the moderated mediation model of this study suggests that learning-related subjective well-being is jointly influenced by Machiavellianism, subjective family economic level, and gratitude. These aspects should be taken into consideration to achieve the best intervention effect and ultimately improve the senior high school students’ learning-related subjective well-being, which could help in the implementation of the “Double Reduction Policy.”

## Data availability statement

The raw data supporting the conclusions of this article will be made available by the authors, without undue reservation.

## Ethics statement

The studies involving human participants were reviewed and approved by the Research Ethical Committee in Zhengzhou University (Reference Number: 305). Written informed consent to participate in this study was provided by the participants’ legal guardian/next of kin.

## Author contributions

MY: research design and protocol and raw manuscript. CQ: research protocol and data analysis. HG, XG, and KT: data collection and analysis. GW: research design and manuscript revisions and corrections. All authors contributed to the article and approved the submitted version.

## Funding

This work was supported by 2020 Western Region/Local Cooperation Project of the China Scholarship Council (CSC) [Granted Number: 202008410388], Henan postdoctoral research start-up project [grant number: 58], Henan Philosophy and Social Science Foundation [grant number: 2019CJY038], and Cultivation plan of excellent young scientific research team in Humanities and Social Sciences of Zhengzhou University [grant number: 6]. But the funding sources had no involvement in study design, in the collection, analysis and interpretation of data, in the writing of the report, and in the decision to submit the article for publication.

## Conflict of interest

The authors declare that the research was conducted in the absence of any commercial or financial relationships that could be construed as a potential conflict of interest.

## Publisher’s note

All claims expressed in this article are solely those of the authors and do not necessarily represent those of their affiliated organizations, or those of the publisher, the editors and the reviewers. Any product that may be evaluated in this article, or claim that may be made by its manufacturer, is not guaranteed or endorsed by the publisher.

## References

[ref1] Aghababaei N. Błachnio A. (2015). Well-being and the dark triad. Personal. Individ. Differ. 86, 365–368. doi: 10.1016/j.paid.2015.06.043

[ref2] Aghababaei N. Błachnio A. Aminikhoo M. (2018). The relations of gratitude to religiosity, well-being, and personality. Mental Health Relig. Cult. 21, 408–417. doi: 10.1080/13674676.2018.1504904

[ref3] Agnew R. (1992). Foundation for a general strain theory of crime and delinquency. Criminology 30, 47–88. doi: 10.1111/j.1745-9125.1992.tb01093.x

[ref4] Alkozei A. Smith R. Killgore W. D. (2018). Gratitude and subjective wellbeing: a proposal of two causal frameworks. J. Happiness Stud. 19, 1519–1542. doi: 10.1007/s10902-017-9870-1

[ref5] Anderman E. M. (2020). Achievement motivation theory: balancing precision and utility. Contemp. Educ. Psychol. 61:101864. doi: 10.1016/j.cedpsych.2020.101864

[ref6] Andrews F. M. McKennell A. C. (1980). Measures of self-reported well-being: their affective, cognitive, and other components. Soc. Indic. Res. 8, 127–155. doi: 10.1007/BF00286474

[ref901] Arathy P. Sampada K. Hansika K. Aneree P. (2021). Gratitude blindness: How does the Dark Triad experience gratitude?. Pers. Individ. Differ. doi: 10.1016/j.paid.2020.110309, PMID: 31107052

[ref7] Asici E. Sari H. I. (2021). A proposed model to explain happiness in college students: the roles of perceived parenting styles, emotional self-efficacy, and forgiveness. J. Adult Dev. 28, 332–345. doi: 10.1007/s10804-021-09378-0

[ref8] Belmi P. Neale M. A. Reiff D. Ulfe R. (2020). The social advantage of miscalibrated individuals: the relationship between social class and overconfidence and its implications for class-based inequality. J. Pers. Soc. Psychol. 118, 254–282. doi: 10.1037/pspi0000187, PMID: 31107052

[ref9] Bianchi R. Mirkovic D. (2020). Is Machiavellianism associated with depression? A cluster-analytic study. Personal. Individ. Differ. 152:109594. doi: 10.1016/j.paid.2019.109594

[ref10] Birkás B. Pátkai G. Csathó Á. (2020). The mediating role of the dark triad between life history strategy and perceived stress factors. Psychol. Rep. 123, 252–265. doi: 10.1177/0033294118818095, PMID: 30572798

[ref11] Bloxsom C. A. Firth J. Kibowski F. Egan V. Sumich A. L. Heym N. (2021). Dark shadow of the self: how the dark triad and empathy impact parental and intimate adult attachment relationships in women. Forensic Sci. Int. Mind Law 2:100045. doi: 10.1016/j.fsiml.2021.100045

[ref12] Brandt N. D. Lechner C. M. Tetzner J. Rammstedt B. (2020). Personality, cognitive ability, and academic performance: differential associations across school subjects and school tracks. J. Pers. 88, 249–265. doi: 10.1111/jopy.12482, PMID: 31009081

[ref13] Cai P. Zuo S. Wang F. Huang N. Xu Y. (2017). The effect of the dark triad personality on Teenagers' orientations to happiness and subject well-being. Chin. J. Spec. Educ. 12, 60–67. doi: 10.3969/j.issn.1007-3728.2017.12.009

[ref14] Canli T. (2004). Functional brain mapping of extraversion and neuroticism: learning from individual differences in emotion processing. J. Pers. 72, 1105–1132. doi: 10.1111/j.1467-6494.2004.00292.x, PMID: 15509278

[ref15] Canli T. Sivers H. Whitfield S. L. Gotlib I. H. Gabrieli J. D. (2002). Amygdala response to happy faces as a function of extraversion. Science 296:2191. doi: 10.1126/science.1068749, PMID: 12077407

[ref16] Cheng Q. Li X. (2010). Dissociation phenomena of implicit self-esteem and explicit self-esteem in Machiavellian individuals. Chin. J. Health Psychol. 18, 1344–1345. doi: 10.13342/j.cnki.cjhp.2010.11.035

[ref17] Chopik W. J. Newton N. J. Ryan L. H. Kashdan T. B. Jarden A. J. (2019). Gratitude across the life span: age differences and links to subjective well-being. J. Posit. Psychol. 14, 292–302. doi: 10.1080/17439760.2017.1414296, PMID: 31105762 PMC6519723

[ref18] Christie R. Geis F. L. (1970). Studies in Machiavellianism. New York: Academic Press.

[ref19] Cohen A. (2016). Are they among us? A conceptual framework of the relationship between the dark triad personality and counterproductive work behaviors (CWBs). Hum. Resour. Manag. Rev. 26, 69–85. doi: 10.1016/j.hrmr.2015.07.003

[ref20] Conger R. D. Donnellan M. B. (2007). An interactionist perspective on the socioeconomic context of human development. Annu. Rev. Psychol. 58, 175–199. doi: 10.1146/annurev.psych.58.110405.085551, PMID: 16903807

[ref21] Crysel L. C. Crosier B. S. Webster G. D. (2013). The dark triad and risk behavior. Personal. Individ. Differ. 54, 35–40. doi: 10.1016/j.paid.2012.07.029

[ref22] Cureu P. L. Coman A. D. Panchenko A. Fodor O. C. Raiu L. (2021). Death anxiety, death reflection and interpersonal communication as predictors of social distance towards people infected with covid-19. Curr. Psychol. 38, 1–14. doi: 10.1007/s12144-020-01171-8, PMID: 33686325 PMC7930891

[ref23] Daniël L. (2022). Sample size justification. Collabra. Psychology 8:33267. doi: 10.1525/collabra.33267

[ref24] Deng Y. Zhu Y. Li Y. Liu X. (2016). Effects of gratitude-based intervention on aggression and subjective well-being in violent criminals. Chin. J. Clin. Psych. 24, 368–372. doi: 10.16128/j.cnki.1005-3611.2016.02.042

[ref905] Diener E. Ryan K. (2009). Subjective Well-Being: A general overview. S. Afr. J. Psychol. 39, 391–406. doi: 10.1177/008124630903900402

[ref25] Ding F. Zhao H. (2018). Is the individual subjective well-being of gratitude stronger? A meta-analysis. Adv. Psychol. Sci. 26, 1749–1763. doi: 10.3724/SP.J.1042.2018.01749

[ref26] Duncan R. J. McClelland M. M. Acock A. C. (2017). Relations between executive function, behavioral regulation, and achievement: moderation by family income. J. Appl. Dev. Psychol. 49, 21–30. doi: 10.1016/j.appdev.2017.01.004

[ref27] Figueredo A. J. Vásquez G. Brumbach B. H. Schneider S. M. Sefcek J. A. Tal I. R.. (2006). Consilience and life history theory: from genes to brain to reproductive strategy. Dev. Rev. 26, 243–275. doi: 10.1016/j.dr.2006.02.002

[ref28] Fredrickson B. L. Joiner T. (2002). Positive emotions trigger upward spirals toward emotional well-being. Psychol. Sci. 13, 172–175. doi: 10.1111/1467-9280.00431, PMID: 11934003

[ref29] Froh J. J. Yurkewicz C. Kashdan T. B. (2008). Gratitude and subjective well-being in early adolescence: examining gender differences. J. Adolesc. 32, 633–650. doi: 10.1016/j.adolescence.2008.06.006, PMID: 18760465

[ref30] Gan X. (2010). Compiling and primary applying of learning-related subjective well-being for junior high school students. Chin. J. School Health 31, 1190–1192. doi: 10.16835/j.cnki.1000-9817.2010.10.018

[ref31] Geng Y. Chang G. Li L. Sun Q. Huang J. (2014). Review on the studies of Machiavellianism trait. Chin. J. Clin. Psych. 22, 816–820. doi: 10.16128/j.cnki.1005-3611.2014.05.058

[ref32] Geng Y. Qin B. Xia D. Han X. (2011). Relations between sophistication and prosocial behavior in adolescents. Chin. J. School Health 32, 44–46. doi: 10.16835/j.cnki.1000-9817.2011.01.021

[ref33] Geng Y. Sun Q. Huang J. Zhu Y. Han X. (2015). Dirty dozen and short dark triad: a Chinese validation of two brief measures of the dark triad. Chin. J. Clin. Psych. 23, 246–250. doi: 10.16128/j.cnki.1005-3611.2015.02.013

[ref970] Gondal M. U. Makhdoom I. F. Malik N. I. (2020). Moderating role of trust within the relationship between machiavellianism and marital Satisfaction. FWU J. Soc. Sci. 14, 28–36., PMID: 16412608

[ref34] Gruenewald T. L. Kemeny M. E. Aziz N. (2006). Subjective social status moderates cortisol responses to social threat. Brain Behav. Immun. 20, 410–419. doi: 10.1016/j.bbi.2005.11.005, PMID: 16412608

[ref35] Hayes A. F. (2017). Introduction to mediation, moderation, and conditional process analysis: A regression-based approach. New York: Guilford publications.

[ref36] He A. Hui Q. Liu H. (2015). Relationship between social support and loneliness in undergraduates: the mediating role of gratitude. Chin. J. Clin. Psych. 23, 150–153. doi: 10.16128/j.cnki.1005-3611.2015.01.034

[ref37] He A. M. Liu H. S. Hui Q. P. (2013). An experimental research on implicit and explicit gratitude of undergraduates. Psychol. Dev. Educ. 45, 22–30. doi: 10.16187/j.cnki.issn1001-4918.2013.01.011

[ref960] He Q. Wang Y. Xing Y. Yu Y. (2018). Dark personality, interpersonal rejection, and marital stability of Chinese couples: An actor–partner interdependence mediation model. Pers. Individ. Differ. 134, 232–238. doi: 10.1016/j.paid.2018.06.003

[ref38] Hou J. (2018). A follow-up study of the impact of achievement goal orientation on academic performance: the mediating effect of gratitude. Chin. J. Spec. Educ. 12, 69–74. doi: 10.3969/j.issn.1007-3728.2018.12.012

[ref39] Hou J. Zou H. Li X. (2009). The characteristics of the family environment and its influence on the life satisfaction of migrant children. Psychol. Dev. Educ. 25, 78–85.

[ref40] House J. S. Lantz P. M. Herd P. (2005). Continuity and change in the social stratification of aging and health over the life course: evidence from a nationally representative longitudinal study from 1986 to 2001/2002 (Americans'Changing lives study). J. Gerontol Series B Psychol. Sci. Soc. Sci. 60, S15–S26. doi: 10.1093/geronb/60.Special_Issue_2.S15 16251586

[ref41] Hu Y. Lan X. (2022). A comprehensive and person-centered view of the association between the dark triad and youth mental health. Front. Psych. 13:900354. doi: 10.3389/fpsyt.2022.900354, PMID: 35845457 PMC9279695

[ref42] Jonason P. K. Duineveld J. J. Middleton J. P. (2015). Pathology, pseudopathology, and the dark triad of personality. Personal. Individ. Differ. 78, 43–47. doi: 10.1016/j.paid.2015.01.028

[ref43] Joshanloo M. (2021). Conceptions of happiness mediate the relationship between the dark triad and well-being. Front. Psychol. 12:643351. doi: 10.3389/fpsyg.2021.643351, PMID: 34045993 PMC8144451

[ref44] Kämmerle M. Unterrainer H. F. Dahmen-Wassenberg P. Fink A. Kapfhammer H. P. (2014). Dimensions of religious/spiritual well-being and the dark triad of personality. Psychopathology 47, 297–302. doi: 10.1159/000358563, PMID: 24852674

[ref45] Lechner C. M. Bender J. Brandt N. D. Rammstedt B. (2021). Two forms of social inequality in Students' socio-emotional skills: do the levels of big five personality traits and their associations with academic achievement depend on parental socioeconomic status? Front. Psychol. 12:679438. doi: 10.3389/fpsyg.2021.679438, PMID: 34367000 PMC8335486

[ref46] Li Y. (2020). Prevalence of suicidal behaviors among primary and middle school students and its relationship with academic burden in Henan Province. Chinese. J. Sch. Health 41, 539–542. doi: 10.16835/j.cnki.1000-9817.2020.04.016

[ref47] Li H. Zhang W. (2014). Peer support: easing up the relationship between Adolescents' learning pressure and depression. Chin. J. Spec. Educ. 10, 87–91. doi: 10.3969/j.issn.1007-3728.2014.10.015

[ref48] Li Z. Zhou F. (2018). Effects of implicit and explicit gratitude on subjective well-being among college students. Chin. J. Clin. Psych. 26, 811–813. doi: 10.16128/j.cnki.1005-3611.2018.04.040

[ref49] Liao H. (2013). The relationship between learning burnout and academic stress in middle school. Modern Prev. Med. 40, 2664–2667.

[ref50] Lorant V. Deliège D. Eaton W. Robert A. Philippot P. Ansseau M. (2003). Socioeconomic inequalities in depression: a meta-analysis. Am. J. Epidemiol. 157, 98–112. doi: 10.1093/aje/kwf182, PMID: 12522017

[ref51] Luo L. Zhou T. (2015). The relationship between gratitude and subjective well-being for middle-school students: the mediation role of anti-frustration ability and social support. Psychol. Dev. Educ. 31, 467–474. doi: 10.16187/j.cnki.issn1001-4918.2015.04.11

[ref52] Luthar S. S. Latendresse S. J. (2005). Children of the affluent: challenges to well-being. Curr. Dir. Psychol. Sci. 14, 49–53. doi: 10.1111/j.0963-7214.2005.00333.x, PMID: 17710193 PMC1948879

[ref53] Ma Y. Liu D. (2005). Middle school Students' learning-related subjective well-being and its major influencing factors. Psychol. Dev. Educ. 1, 74–79.

[ref54] Ma M. Liu Y. Zhao N. (2021). The dark triad traits and the prediction of Eudaimonic well-being. Front. Psychol. 12, 693778–693778. doi: 10.3389/fpsyg.2021.693778 34803793 PMC8602073

[ref951] Manstead A. (2018). The psychology of social class: How socioeconomic status impacts thought, feelings, and behaviour. Br. J. Soc. Psychol. 57, 267–291. doi: 10.1111/bjso.12251 29492984 PMC5901394

[ref55] McCullough M. E. Kilpatrick S. D. Emmons R. A. Larson D. B. (2001). Is gratitude a moral affect? Psychol. Bull. 127, 249–266. doi: 10.1037/0033-2909.127.2.249, PMID: 11316013

[ref56] McDonald M. M. Donnellan M. B. Navarrete C. D. (2012). A life history approach to understanding the dark triad. Personal. Individ. Differ. 52, 601–605. doi: 10.1016/j.paid.2011.12.003

[ref57] McGonagle K. A. Kessler R. C. (1990). Chronic stress, acute stress, and depressive symptoms. Am. J. Community Psychol. 18, 681–706. doi: 10.1007/BF00931237 2075897

[ref59] Mullen B. Symons C. Hu L. T. Salas E. (1989). Group size, leadership behavior, and subordinate satisfaction. J. Gen. Psychol. 116, 155–170. doi: 10.1080/00221309.1989.9711120

[ref61] Muris P. Meesters C. Timmermans A. (2013). Some youths have a gloomy side: correlates of the dark triad personality traits in non-clinical adolescents. Child Psychiatry Hum. Dev. 44, 658–665. doi: 10.1007/s10578-013-0359-9, PMID: 23334267

[ref62] Navarro-Carrillo G. Alonso-Ferres M. Moya M. Valor-Segura I. (2020). Socioeconomic status and psychological well-being: revisiting the role of subjective socioeconomic status. Front. Psychol. 11:1303. doi: 10.3389/fpsyg.2020.01303, PMID: 32587560 PMC7298147

[ref63] Nielsen I. Smyth R. Liu Y. (2011). The moderating effects of demographic factors and hukou status on the job satisfaction–subjective well-being relationship in urban China. Int. J. Hum. Resour. Manag. 22, 1333–1350. doi: 10.1080/09585192.2011.559103

[ref64] Peng X. Cai T. Gui T. Fu J. (2021). Moderating effect of psychological sushi on relationship between study stress and suicidal ideation in adolescents. Chin. Ment. Health J. 35, 919–924. doi: 10.3969/j.issn.1000-6729.2021.11.006

[ref65] Podsakoff P. M. MacKenzie S. B. Lee J. Y. Podsakoff N. P. (2003). Common method biases in behavioral research: a critical review of the literature and recommended remedies. J. Appl. Psychol. 88, 879–903. doi: 10.1037/0021-9010.88.5.879, PMID: 14516251

[ref67] Qin F. (2013). Moral psychological studies on Machiavellianism. Psychol. Explor. 33, 15–21. doi: 10.3969/j.issn.1003-5184.2013.01.003

[ref68] Rauthmann J. F. (2011). Acquisitive or protective self-presentation of dark personalities? Associations among the dark triad and self-monitoring. Personal. Individ. Differ. 51, 502–508. doi: 10.1016/j.paid.2011.05.008

[ref69] Rehman U. Nabi A. Shahnawaz M. G. (2018). Machiavellianism, influential tactics and well-being among Indian college students. Psychol. Stud. 63, 42–51. doi: 10.1007/s12646-018-0443-0

[ref70] Shen C. Li H. Song W. Jiang S. Bao J. (2021). The effect of stressful life events on non-suicidal self-injury in Chinese adolescents: the moderating role of positive youth development. Chin. J. Clin. Psych. 29, 483–488. doi: 10.16128/j.cnki.1005-3611.2021.03.008

[ref71] Sherry S. B. Hewitt P. L. Besser A. Flett G. L. Klein C. (2006). Machiavellianism, trait perfectionism, and perfectionistic self-presentation. Personal. Individ. Differ. 40, 829–839. doi: 10.1016/j.paid.2005.09.010

[ref72] Shi L. Chen Y. Hou X. Gao F. (2013). Social economic status and study engagement: the mediating effects of academic self-efficacy among junior high school. Psychol. Dev. Educ. 29, 71–78. doi: 10.16187/j.cnki.issn1001-4918.2013.01.006

[ref73] Singh-Manoux A. Marmot M. G. Adler N. E. (2005). Does subjective social status predict health and change in health status better than objective status? Psychosom. Med. 67, 855–861. doi: 10.1097/01.psy.0000188434.52941.a0, PMID: 16314589

[ref74] Song L. Liu R. Li Y. Gao Z. Li W. (2010). The influence of social support, learning view and self-efficacy on middle school Students' learning-related subjective well-being. Psychol. Dev. Educ. 26, 282–287. doi: 10.16187/j.cnki.issn1001-4918.2010.03.012

[ref75] Suh E. Diener E. Fujita F. (1996). Events and subjective well-being: only recent events matter. J. Pers. Soc. Psychol. 70, 1091–1102. doi: 10.1037/0022-3514.70.5.1091, PMID: 8656337

[ref76] Tan J. J. Kraus M. W. Carpenter N. C. Adler N. E. (2020). The association between objective and subjective socioeconomic status and subjective well-being: a meta-analytic review. Psychol. Bull. 146, 970–1020. doi: 10.1037/bul0000258, PMID: 33090862

[ref77] Tenenbaum H. R. Ruck M. D. (2007). Are teachers' expectations different for racial minority than for European American students? A meta-analysis. J. Educ. Psychol. 99, 253–273. doi: 10.1037/0022-0663.99.2.253

[ref78] Thompson M. N. (2013). Career barriers and coping efficacy among native American students. J. Career Assess. 21, 311–325. doi: 10.1177/1069072712471501

[ref80] Van Groningen A. J. Grawitch M. J. Lavigne K. N. Palmer S. N. (2021). Every cloud has a silver lining: Narcissism’s buffering impact on the relationship between the dark triad and well-being. Personal. Individ. Differ. 171:110549. doi: 10.1016/j.paid.2020.110549

[ref81] Walberg H. J. Tsai S. L. (1983). Matthew effects in education. Am. Educ. Res. J. 20, 359–373.

[ref82] Wang Q. Liu X. (2021). Peer victimization and nonsuicidal self-injury among Chinese left-behind children: the moderating roles of subjective socioeconomic status and social support. J. Interpers. Violence 36, 11165–11187. doi: 10.1177/0886260519897346, PMID: 31916475

[ref83] Wanous J. P. Reichers A. E. (1996). Estimating the reliability of a single-item measure. Psychol. Rep. 78, 631–634. doi: 10.2466/pr0.1996.78.2.631

[ref84] Wanous J. P. Reichers A. E. Hudy M. J. (1997). Overall job satisfaction: how good are single-item measures? J. Appl. Psychol. 82, 247–252. doi: 10.1037/0021-9010.82.2.247, PMID: 9109282

[ref85] Watson D. Clark L. A. (1992). On traits and temperament: general and specific factors of emotional experience and their relation to the five-factor model. J. Pers. 60, 441–476. doi: 10.1111/j.1467-6494.1992.tb00980.x, PMID: 1635050

[ref86] Watson D. Wiese D. Vaidya J. Tellegen A. (1999). The two general activation systems of affect: structural findings, evolutionary considerations, and psychobiological evidence. J. Pers. Soc. Psychol. 76, 820–838. doi: 10.1037/0022-3514.76.5.820

[ref950] Wei C. Wu H. T. Kong X. N. Wang H. (2011). Revision of Gratitude Questionnaire-6 in Chinese adolescent and its validity and reliability. Chin. J. Sch. Health. 32, 1201–1202. doi: 10.16835/j.cnki.1000-9817.2011.10.016

[ref87] Wood A. M. Froh J. J. Geraghty A. W. (2010). Gratitude and well-being: a review and theoretical integration. Clin. Psychol. Rev. 30, 890–905. doi: 10.1016/j.cpr.2010.03.005, PMID: 20451313

[ref88] Xie X. Zhang W. Yu C. Zhou Y. Ye H. Chen J. (2013). Adolescent materialism and well-being: a mediating effect of gratitude. J. Psychol. Sci. 36, 638–646. doi: 10.16719/j.cnki.1671-6981.2013.03.011

[ref89] Yan W. Zhang L. Li W. You X. Kong F. (2022). Associations of family subjective socioeconomic status with hedonic and eudaimonic well-being in emerging adulthood: a daily diary study. Soc. Sci. Med. 298:114867. doi: 10.1016/j.socscimed.2022.114867, PMID: 35276625

[ref90] Yang M. Guo H. Chu M. Leng C. Qu C. Tian K.. (2022). Sex differences in traditional school bullying perpetration and victimization among adolescents: a chain-mediating effect. Int. J. Environ. Res. Public Health 19:9525. doi: 10.3390/ijerph19159525, PMID: 35954881 PMC9368119

[ref91] Yang X. Qi Y. Shen Z. Han B. Meng B. (2015). Family socioeconomic status, vocational value on subjective well-being in medical students. Chin. J. Clin. Psych. 23, 154–158. doi: 10.16128/j.cnki.1005-3611.2015.01.035

[ref92] Yang K. Yan W. Jia N. Wang Q. Kong F. (2021). Longitudinal relationship between trait gratitude and subjective well-being in adolescents: evidence from the bi-factor model. J. Posit. Psychol. 16, 802–810. doi: 10.1080/17439760.2020.1818812

[ref93] Yang Q. Ye B. (2014). The effect of gratitude on Adolescents' life satisfaction: the mediating role of perceived social support and the moderating role of stressful life events. J. Psychol. Sci. 37, 610–616. doi: 10.16719/j.cnki.1671-6981.2014.03.018

[ref94] Yang M. Zhu X. Sai X. Zhao F. Wu H. Geng Y. (2019). The dark triad and sleep quality: mediating role of anger rumination. Personal. Individ. Differ. 151:109484. doi: 10.1016/j.paid.2019.06.027

[ref95] Yao B. Zhou X. Quan L. Zhang L. (2015). The investigating of situation and factors of adolescents’ ideals in China. Psychol. Dev. Educ. 31, 71–77. doi: 10.16187/j.cnki.issn1001-4918.2015.01.10

[ref96] Yu Y. Zheng X. (2005). Psychological stress and influencing factors of graduate students. Psychol. Dev. Educ. 3, 99–103.

[ref97] Zhang L. Hou X. (2010). Gratitude: concept, measurement and related studies. Psychol. Sci. 33, 393–395. doi: 10.16719/j.cnki.1671-6981.2010.02.031

[ref98] Zhang Y. Yuan B. Wang K. Shen T. (2021). The effect of impulsivity traits on senior high school students’ suicidal ideation: the role of campus exclusion and sense of life meaning. Stud. Psychol. Behav. 19, 89–95.

